# Hypotony-Free Closure of Infusion Sclerotomy Using a Slit-Modified Trocar in 23-Gauge Vitrectomy for Proliferative Diabetic Retinopathy

**DOI:** 10.3390/bioengineering13050580

**Published:** 2026-05-19

**Authors:** Goran Marić, Danny A. Mammo, Ante Vukojević, Armin Kasumović, Mia Zorić Geber, Katia Novak Lauš, Rašeljka Tadić, Tena Križ, Marin Radmilović, Zoran Vatavuk

**Affiliations:** 1 Department of Ophthalmology, University Hospital Center Sestre Milosrdnice, Vinogradska Cesta 29, 10000 Zagreb, Croatia; ante.vukojevic1@gmail.com (A.V.); katia@midij-com.hr (K.N.L.);; 2 Cole Eye Institute, Cleveland Clinic, Cleveland, OH 44195, USA

**Keywords:** vitrectomy, 23-gauge, sclerotomy, hypotony, trocar, intraocular pressure, vitreoretinal surgery, biomedical device

## Abstract

Purpose: The aim of this study is to describe a slit-modified 23-gauge infusion trocar designed to enable early postoperative hypotony-free sclerotomy closure by allowing scleral suturing prior to complete trocar removal, and to report initial clinical outcomes in eyes with proliferative diabetic retinopathy with or without vitreous hemorrhage (PDR + H and PDR). Methods: A standard 23-gauge metallic (titanium) trocar was modified by creating a longitudinal slit that permitted passage of a suture needle while the trocar remained partially engaged within the scleral tunnel. At the end of pars plana vitrectomy, a transscleral suture was placed through the slit with the knot prepared prior to trocar removal, followed by simultaneous trocar extraction and suture tightening. Eighteen consecutive patients undergoing vitrectomy for PDR (fourteen with vitreous hemorrhage [PDR + H]; four without) were included. Intraocular pressure (IOP) was recorded preoperatively, immediately after sclerotomy closure (postoperative baseline), and at 8 and 24 h postoperatively. The study was designed as an exploratory pilot feasibility and safety evaluation of a slit-modified infusion trocar in 23-gauge vitrectomy. The primary outcomes were postoperative IOP stability and wound leakage. Secondary outcomes included early hypotony, postoperative hemorrhage, choroidal effusion, and the need for additional suturing. Results: All procedures were completed without intraoperative complications. The mean IOP was 14.83 ± 2.50 mmHg preoperatively, 13.33 ± 1.53 mmHg immediately after closure, 14.17 ± 3.01 mmHg at 8 h, and 15.17 ± 1.79 mmHg at 24 h. No cases of wound leakage or early postoperative hypotony were observed in either subgroup. One eye exhibited a transient IOP increase at 8 h; no choroidal effusion, postoperative hemorrhage, or need for secondary suturing occurred. Endotamponade consisted of balanced salt solution (BSS) in eight eyes, SF6 in seven eyes, silicone oil in two eyes, and air in one eye. Conclusions: The slit-modified infusion trocar enables secure, hypotony-free closure of the infusion sclerotomy by eliminating the open-wound interval during trocar removal. This simple biomedical device modification provides stable early postoperative IOP across different tamponade agents and appears safe and feasible in high-risk eyes with PDR.

## 1. Introduction

Microincision vitrectomy surgery (MIVS) has become the standard approach for the management of vitreoretinal diseases, offering reduced surgical trauma, faster recovery, and improved patient comfort. Although the field has progressively shifted toward smaller gauge systems (25- and 27-gauge), 23-gauge vitrectomy continues to be widely performed in routine clinical practice, particularly in complex cases requiring greater instrument rigidity, enhanced flow rates and improved surgical control [1,2].

In contrast, smaller-gauge systems (25- and 27-gauge) rarely require suturing because of the intrinsic self-sealing properties of the sclera at these dimensions, sometimes requiring only simple hydration at the end of surgery. Consequently, the problem of transient wound instability during port removal is largely mitigated in very-small-gauge surgery [3,4,5]. This makes the infusion sclerotomy in 23-gauge vitrectomy a uniquely relevant target for innovations aimed at improving wound closure and pressure stability.

However, despite advances in trocar–cannula systems and self-sealing sclerotomies, early postoperative hypotony remains a clinically relevant complication. In particular, the infusion sclerotomy represents a critical site where transient loss of wound integrity during trocar removal may result in abrupt intraocular pressure (IOP) decrease, fluid egress, and wound instability.

Early postoperative hypotony has been associated with a range of complications, including choroidal effusion, postoperative hemorrhage, hypotony maculopathy, and increased risk of endophthalmitis. These risks are especially pronounced in myopic eyes, younger patients, and procedures without tamponade. They are further increased in eyes with extensive peripheral vitreous removal or prior vitrectomy [6,7,8,9] and especially in eyes with proliferative diabetic retinopathy (PDR), where fragile neovascular tissue and the absence of a stabilizing vitreous scaffold predispose patients to postoperative bleeding and pressure-related complications. Eyes with proliferative diabetic retinopathy are particularly susceptible to early postoperative hypotony due to extensive membrane dissection and prolonged surgical manipulation. Because the macular structure and perfusion are often already impaired in PDR, hypotony-related maculopathy may have a disproportionate impact on visual outcomes in this population. Consequently, secure and immediate closure of the infusion sclerotomy is of particular importance in this high-risk population [6,10].

Current strategies to address wound leakage include oblique or tunneled sclerotomies, valved cannula systems, wound hydration or cauterization, early preplaced suture and selective suturing of leaking ports [5,11,12,13,14,15,16,17,18]. Nevertheless, a fundamental limitation persists: suturing is typically performed after trocar removal, creating a brief but critical “open-wound interval” during which intraocular pressure may drop and ocular contents may shift. While this interval is often short, its mechanical and physiological consequences may be clinically significant, particularly in eyes prone to hemorrhagic or hypotony-related complications.

From an engineering perspective, an ideal solution would enable secure sclerotomy closure before complete trocar withdrawal. This would preserve wound architecture and maintain IOP throughout the process. To the best of our knowledge, although several techniques have been described, no standard trocar system with an integrated or preloaded suture mechanism for controlled closure of the final sclerotomy—while the instrument remains partially engaged within the scleral tunnel and continues to provide a sealing function—has been described. More broadly, translational biomedical engineering increasingly emphasizes clinically targeted device-level innovations capable of improving procedural safety, workflow reproducibility, and postoperative outcomes through relatively simple mechanical modifications of existing medical technologies. Similar translational principles have been highlighted across other medical device fields, including biosensor development and wearable and sensor-based healthcare technologies [19,20].

Here, we describe a simple modification of a standard 23-gauge infusion trocar, in which a longitudinal slit allows for the passage of a suture needle while the trocar remains partially in situ. This design enables transscleral suturing prior to trocar removal, eliminating the open-wound interval and facilitating hypotony-free closure of the infusion sclerotomy. We further report initial clinical outcomes in a consecutive series of patients undergoing vitrectomy for proliferative diabetic retinopathy, with and without vitreous hemorrhage, to assess the safety, feasibility, and early pressure stability of this biomedical device innovation.

## 2. Materials and Methods

### 2.1. Trocar Design

**Figure 1:**
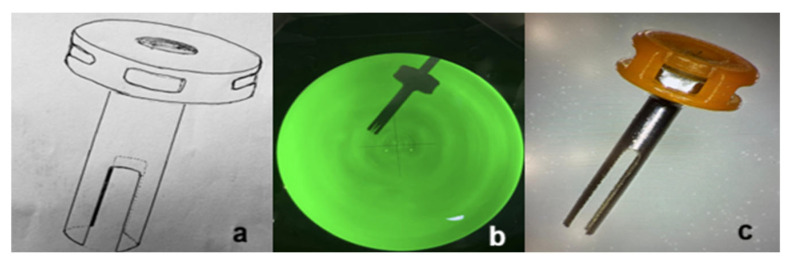
Slit-trocar. (**a**) Idea—scheme, (**b**) trocar and partially pulled off stylet—view under the industrial microscope, (**c**) slit-modified 23G trocar—prepared for sterilization and use.

A standard 23-gauge metallic (titanium) trocar (Alcon, Fort Worth, TX, USA) was modified by creating a longitudinal slit using a wire EDM (electrical discharge machining) process. It was created ensuring smooth edges and preservation of structural integrity, extending to a length of 2 mm (from the external opening to approximately half of the trocar shaft length), while the width was approximately 0.3 mm. The slit length was intentionally limited to approximately half of the trocar shaft length in order to preserve structural stability and sealing properties. The dimensions correspond to the diameter of the 7-0 polyglactin absorbable suture needle (7-0 polyglactin 910 suture, Vicryl; Ethicon, Inc., Raritan, NJ, USA), enabling controlled needle passage while maintaining effective sealing of the trocar within the scleral tunnel (Figure 1).

Following industrial modification, all modified trocars were sterilized using ethylene oxide in accordance with standard medical device sterilization protocols prior to clinical use. No structural deformation or compromise of trocar stability was observed during repeated intraoperative use or subsequent industrial microscope inspection.

### 2.2. Surgical Technique

**Figure 2:**
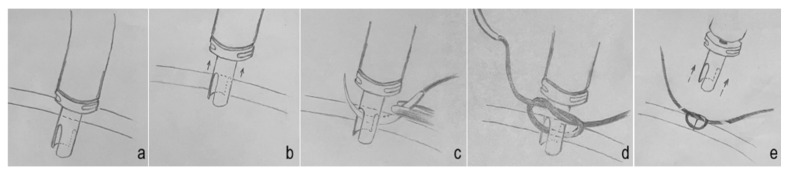
Slit-trocar—surgical technique diagram. (**a**) Transscleral position of the slit-trocar at the end of vitrectomy, (**b**) position of the slit-trocar after stopping of endotamponade agent and partial withdrawal, (**c**) suture needle inside the trocar longitudinal slit and passed through both sides of the sclerotomy, (**d**) preparation of the knot while trocar continues to seal the sclerotomy, (**e**) simultaneous removal of slit-trocar and suture knot tightening, resulting in immediate sclerotomy closure and stable IOP.

At the end of pars plana vitrectomy (Figure 2a), the infusion cannula was partially withdrawn, leaving the trocar still engaged within the scleral tunnel. Immediately prior to partial withdrawal of the trocar from the sclerotomy, infusion of the endotamponade agent was temporarily stopped to eliminate any theoretical risk of fluid/tamponade agent egress into the layers of the ocular wall (Figure 2b). While the trocar remained in this semi-withdrawn position, a suture needle was passed transsclerally on one side of the sclerotomy, then through the longitudinal slit of the trocar, and subsequently through the sclera on the opposite side of the wound. The trocar continued to seal the sclerotomy during this step (Figure 2c). A knot was prepared externally (Figure 2d). The trocar was then removed while simultaneous tightening of the suture was performed (Figure 2e), resulting in immediate closure of the sclerotomy without tamponade agent egress or pressure drop (Video S1). No structural deformation or compromise of mechanical stability of the trocar was observed during repeated intraoperative use.

### 2.3. Patients and Outcomes

Eighteen consecutive patients undergoing 23-gauge pars plana vitrectomy for proliferative diabetic retinopathy were included: fourteen eyes with PDR complicated by vitreous hemorrhage (PDR + H) and four eyes with PDR without hemorrhage. All surgeries were performed by two vitreoretinal surgeons in the early phase of independent practice in the morning shift to avoid the impact of daily physiological IOP fluctuations [21]. IOP was measured using a Perkins handheld applanation tonometer in the supine position, as required for immediate postoperative assessment. This method is considered reliable for supine intraocular pressure measurement in the immediate postoperative setting. Measurements were obtained at four time points: preoperatively, immediately after sclerotomy closure (postoperative baseline), and at 8 and 24 h postoperatively. The study was designed as an exploratory pilot feasibility and safety evaluation of a slit-modified infusion trocar in 23-gauge vitrectomy. Primary outcomes were postoperative IOP stability and wound leakage (Seidel test). Secondary outcomes included early hypotony, choroidal effusion, postoperative hemorrhage, and the need for additional suturing. Device-related safety was additionally assessed through intraoperative observation of trocar stability, structural integrity, and the absence of device-related complications. Given the exploratory nature of this pilot study, only descriptive statistics were performed. Endotamponade (BSS, SF6, silicone oil or air), lens status (phakic/pseudophakic), and preoperative anti-VEGF use were documented.

## 3. Results

All eighteen surgeries were completed without intraoperative complications. Key demographic, biometric, surgical and intraocular pressure (IOP) data are summarized in Table 1.

**Figure 3:**
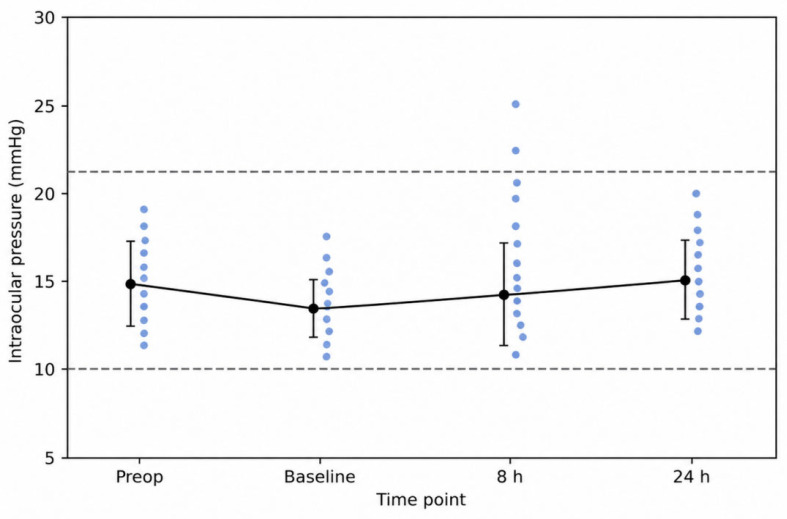
Intraocular pressure curve following hypotony-free sclerotomy closure (*N* = 18). Mean intraocular pressure values (±SD) measured preoperatively, immediately after closure (baseline), and at 8 and 24 h postoperatively. Each blue dot represents an individual eye (*N* = 18). Dashed lines indicate physiological reference range (10–21 mmHg).

Mean preoperative IOP was 14.8 ± 2.5 mmHg. Mean postoperative baseline IOP immediately after sclerotomy closure was 13.3 ± 1.5 mmHg, remaining at 14.2 ± 3.0 mmHg at 8 h and 15.2 ± 1.8 mmHg at 24 h (Figure 3). IOP values remained within the physiological range at all time points.

No eye demonstrated wound leakage on Seidel testing at any time point. No cases of early postoperative hypotony, choroidal effusion, postoperative vitreous hemorrhage, or endophthalmitis were observed. No secondary suturing was required.

Endotamponade consisted of balanced saline solution (BSS) in eight eyes, sulfur hexafluoride (SF6) in seven eyes, silicone oil in two eyes, and air in one eye. Stable early postoperative IOP was observed across different tamponade agents within this exploratory pilot series. One eye (SF6) exhibited a transient IOP increase of 25 mmHg at 8 h, which resolved without sequelae and with no added antiglaucoma medications. Preoperative anti-VEGF had been administered in 6 of 18 eyes.

## 4. Discussion

The present technique is intentionally specific to 23-gauge vitrectomy. In 25-gauge and 27-gauge systems, sclerotomies are typically self-sealing due to the biomechanical properties of the sclera and the smaller incision size, and suturing is rarely required. As a result, the mechanical problem addressed by the current design—namely, the transient open-wound interval during trocar removal—does not usually arise in smaller-gauge surgery. The clinical value of this modification therefore lies precisely in its application to 23-gauge platforms, which continue to be widely used in complex vitreoretinal procedures where greater instrument rigidity, higher flow rates, and more stable fluidics may still provide practical surgical advantages [1,2].

Infusion sclerotomy was selected because it represents the primary source of continuous intraoperative pressure regulation and therefore the most critical site for preventing pressure loss during port removal. The principal advantage of the described technique is the elimination of the unprotected interval between trocar removal and sclerotomy closure. By allowing suture placement while the trocar remains partially within the scleral tunnel, intraocular pressure is maintained and wound architecture preserved, resulting in hypotony-free closure of the infusion port. This was associated with stable IOP values immediately after closure and at 8 and 24 h postoperatively, without wound leakage or hypotony, across both PDR + H and PDR subgroups.

Importantly, stable early postoperative IOP was observed across different endotamponades (BSS, SF6, silicone oil, and air). However, given the small number of cases within individual tamponade subgroups, these observations should be interpreted cautiously and cannot establish tamponade-independent performance. Although the slit may theoretically reduce sealing integrity, no clinically significant gas, silicone oil, or fluid leakage was observed in the present series. Ando et al. identified the absence of tamponade as a significant risk factor for early postoperative hypotony, with gas tamponade cited as protective by promoting sclerotomy closure through surface tension and preventing postoperative leakage [6]. Only one transient IOP elevation occurred, with no hypotony-related or hemorrhagic complications.

This is particularly relevant in proliferative diabetic retinopathy, where early hypotony may precipitate hemorrhage or choroidal complications [6,7,10].

The described approach may be especially beneficial for less experienced vitreoretinal surgeons, for whom precise timing and wound stability during removal of the final trocar may represent a greater technical challenge [22]. By allowing suturing while the trocar remains partially in situ, the technique minimizes the time-dependent loss of intraocular pressure and fluid or tamponade agent egress. This advantage is particularly relevant in eyes with gas tamponade, where rapid escape can occur [7], and in cases using silicone oil, where the lubricating properties of the medium may make conventional suturing more technically challenging [22,23].

Limitations include the small sample size and lack of a comparative control group. Because this was an exploratory pilot study without a control group, the findings should be interpreted as preliminary feasibility and safety data rather than evidence of superiority over conventional sclerotomy closure. Although a direct control group was not included, postoperative hypotony following standard 23-gauge vitrectomy has been reported in the literature, and the absence of hypotony in the present series may therefore be clinically relevant, although this observation should be interpreted with caution given the limited sample size. Further larger comparative studies are warranted to confirm these findings and to better define the clinical impact of this approach.

## 5. Conclusions

The slit-modified 23-gauge trocar appears to be a feasible approach for facilitating transconjunctival sclerotomy suturing and was associated with stable early postoperative IOP in this initial clinical series. This simple biomedical device modification provides stable early postoperative IOP across different tamponade agents and appears to be safe and effective in high-risk eyes with proliferative diabetic retinopathy. The technique offers a simple and reproducible solution for hypotony-free closure that may enhance surgical safety, especially in less experienced hands.

## 6. Patents

The inventors of this modified trocar, Zoran Vatavuk and Goran Marić, currently do not pursue patent protection for this concept in order to facilitate clinical adoption. All drawings are performed by the first author of this article.

## Figures and Tables

**Table 1:** Patient characteristics, surgical details, and intraocular pressure measurements.

No.	Sex	Age (Years)	AL (mm)	Lens Status	Indication	Preop IOP (mmHg)	IOP Baseline (mmHg)	IOP 8 h (mmHg)	IOP 24 h (mmHg)	Tamponade/Complications	Anti-VEGF (1 = YES, 0 = NO)
1	F	63	22.31	P	PDR + H	18	13	12	14	BSS/No	1
2	M	67	23.46	P	PDR + H	11	12	13	15	BSS/No	0
3	M	71	23.8	P	PDR + H	12	11	14	15	SF6/No	0
4	M	77	24.02	P	PDR + H	13	13	12	13	BSS/No	0
5	F	68	22.89	P	PDR + H	17	14	12	16	SF6/No	1
6	M	66	24.77	P	PDR + H	11	12	12	12	BSS/No	0
7	F	57	22.63	P	PDR + H	18	14	13	15	SF6/No	1
8	F	64	23.14	N	PDR + H	16	12	14	16	BSS/No	1
9	M	55	22.98	P	PDR + H	15	12	13	16	SF6/No	0
10	M	66	22.37	P	PDR + H	19	13	25	20	SF6/transitory IOP increase	1
11	M	59	23.06	N	PDR	17	14	13	16	Silicone oil/No	0
12	F	62	23.65	P	PDR	13	11	12	13	Air/No	0
13	M	44	24.03	N	PDR + H	14	16	16	17	Silicone oil/No	0
14	F	56	24.18	P	PDR + H	16	13	14	16	SF6/No	0
15	M	71	25.02	P	PDR	13	15	15	14	BSS/No	0
16	M	55	24.73	N	PDR + H	14	16	16	15	BSS/No	1
17	F	62	23.46	N	PDR	17	14	15	16	BSS/No	0
18	M	58	24.85	P	PDR + H	13	15	14	14	SF6/No	0

Abbreviations: No. = number, F = female, M = male, AL = axial length of the eye, N = natural lens, phakic eye, P = pseudophakic eye, PDR = proliferative diabetic retinopathy, H = hematovitreum, IOP = intraocular pressure, BSS = balanced salt solution, SF6 = sulfur hexafluoride, Anti-VEGF = anti vascular endothelial growth factor.

## Data Availability

The original contributions presented in this study are included in the article. Further inquiries can be directed to the corresponding author(s).

## Supplementary Materials

The following supporting information can be downloaded at https://www.mdpi.com/article/10.3390/bioengineering13050580/s1: Video S1: Notched 23-gauge trocar.

## Abbreviations

The following abbreviations are used in this manuscript:

MIVS	Microincision Vitrectomy Surgery
IOP	Intraocular Pressure
PDR + H	Proliferative Diabetic Retinopathy + Hemorrhage
BSS	Balanced Salt Solution
antiVEGF	Anti-Vascular Endothelial Growth Factor
SF6	Sulfur Hexafluoride

## References

[1] Mohamed S., Claes C., Tsang C.W. (2017). Review of Small Gauge Vitrectomy: Progress and Innovations. J. Ophthalmol..

[2] Ma J., Wang Q., Niu H. (2020). Comparison of 27-Gauge and 25-Gauge Microincision Vitrectomy Surgery for the Treatment of Vitreoretinal Disease: A Systematic Review and Meta-Analysis. J. Ophthalmol..

[3] Caretti L., Pillon G., Verzola G., Monterosso C., Formisano M. (2024). A Prospective Randomized Study Comparing 27-Gauge Vitrectomy to 23-Gauge Vitrectomy for Epiretinal Membranes and Full-Thickness Macular Holes. J. Curr. Ophthalmol..

[4] Xiang W., Fang D., Jiang X., Zhang Z., Xiang C., Huang S., Zhang S., Wei Y. (2023). 27-Gauge vitrectomy vs. 25-gauge vitrectomy in the management of proliferative diabetic retinopathy with preoperative intravitreal injection of conbercept. Exp. Ther. Med..

[5] Rossi T., Querzoli G., Angelini G.B., Pellizzaro C., Santoro V., Rosari G., Parravano M., Steel D.H., Romano M.R. (2025). Comparing the Efficiency of Valved Trocar Cannulas for Pars Plana Vitrectomy. Bioengineering.

[6] Ando T., Terashima H., Fujii K., Yoshida H., Ueda E., Nozaki Y., Shiozaki N., Yaoeda K., Fukuchi T. (2025). Risk factors for hypotony after transconjunctival sutureless vitrectomy. PLoS ONE.

[7] Woo S.J., Park K.H., Hwang J.M., Kim J.H., Yu Y.S., Chung H. (2009). Risk factors associated with sclerotomy leakage and postoperative hypotony after 23-gauge transconjunctival sutureless vitrectomy. Retina.

[8] Byeon S.H., Lew Y.J., Kim M., Kwon O.W. (2008). Wound leakage and hypotony after 25-gauge sutureless vitrectomy: Factors affecting postoperative intraocular pressure. Ophthalmic Surg. Lasers Imaging.

[9] Ouyang X., Han Y., Xie Y., Wu Y., Guo S., Cheng M., Wang G. (2019). The collagen metabolism affects the scleral mechanical properties in the different processes of scleral remodeling. Biomed. Pharmacother..

[10] Lee J.Y., Jeong H.S., Lee D.Y., Sohn H.J., Nam D.H. (2012). Early postoperative intraocular pressure stability after combined 23-gauge sutureless vitrectomy and cataract surgery in patients with proliferative diabetic retinopathy. Retina.

[11] López-Guajardo L., Pareja-Esteban J., Teus-Guezala M.A. (2006). Oblique sclerotomy technique for prevention of incompetent wound closure in transconjunctival 25-gauge vitrectomy. Am. J. Ophthalmol..

[12] Trichonas G., Kaiser P.K. (2014). Wound construction. Dev. Ophthalmol..

[13] Lambat S.P., Khanna N.N., Nangia V.B. (2025). Sclerotomy port suturing in microincisional vitrectomy surgery. Oman J. Ophthalmol..

[14] Lee B.R., Song Y. (2008). Releasable suture technique for the prevention of incompetent wound closure in transconjunctival vitrectomy. Retina.

[15] Arana L.A., Moreira A.T.R., Grandinetti A.A., Moreira H., Moreria C.A. (2019). Novel Vicryl Releasable Suture Technique to Close Leaking Sclerotomies in a Transconjunctival Vitrectomy. Retina.

[16] Savastano A., Crincoli E., Caporossi T., Rizzo C., Savastano M.C., De Vico U., Rizzo S. (2023). New Releasable Nonabsorbable Polypropylene 8.0 Suturing Technique for Sclerotomy Sealing in 23-Gauge Vitrectomy. Retina.

[17] Rizzo S., Pacini B., De Angelis L., Barca F., Savastano A., Giansanti F., Caporossi T. (2022). Intrascleral hydration for 23-Gauge Pars Plana Vitrectomy Sclerotomy Closure. Retina.

[18] Boscia F., Besozzi G., Recchimurzo N., Sborgia L., Furino C. (2011). Cauterization for the prevention of leaking sclerotomies after 23-gauge transconjunctival pars plana vitrectomy: An easy way to obtain sclerotomy closure. Retina.

[19] Pullano S.A., Greco M., Bianco M.G., Foti D., Brunetti A., Fiorillo A.S. (2022). Glucose biosensors in clinical practice: Principles, limits and perspectives of currently used devices. Theranostics.

[20] Mukhopadhyay S.C. (2019). Low-Power Wearable and Wireless Sensors for Advanced Healthcare Monitoring.

[21] Fogagnolo P., Orzalesi N., Ferreras A., Rossetti L. (2009). The circadian curve of intraocular pressure: Can we estimate its characteristics during office hours?. Investig. Ophthalmol. Vis. Sci..

[22] Yeh S., Chan-Kai B.T., Lauer A.K. (2011). Basic training module for vitreoretinal surgery and the Casey Eye Institute Vitrectomy Indices Tool for Skills Assessment. Clin. Ophthalmol..

[23] Chen Y., Kearns V.R., Zhou L., Sandinha T., Lam W.C., Steel D.H., Chan Y.K. (2021). Silicone oil in vitreoretinal surgery: Indications, com-plications, new developments and alternative long-term tamponade agents. Acta Ophthalmol..

